# Tissue Regeneration on Implantoplasty-Treated Implants Using a Citric Acid–Collagen–Magnesium-Based Solution: An In Vitro and In Vivo Study

**DOI:** 10.3390/biomimetics11020116

**Published:** 2026-02-04

**Authors:** Samuel Oliván, Pedro Fernández-Domínguez, Javier Gil, Manuel Fernández-Domínguez

**Affiliations:** 1 Departament Biomaterials, EDE, University of La Salle, c/Gaminedes 11, 28023 Madrid, Spain; doctorolivan@edeformacion.com; 2 Facultad de Odontología, Universidad Camilo José Cela, C/Castillo de Alarcón, 49. Urb. Villafranca del Castillo, 28691 Madrid, Spain; pedro.fernandezd@ucjc.es (P.F.-D.); manuelfdominguez@ucjc.es (M.F.-D.); 3 Bioinspired Oral Biomaterials and Interfaces, Department Ciencia e Ingenieria de Materiales, Escola Enginyeria Barcelona Est., Universitat Politècnica de Catalunya, Av. Eduard Maristany 16, 08019 Barcelona, Spain; 4 Department of Translational Medicine, CEU San Pablo University, Urbanización Montepríncipe, 28925 Madrid, Spain

**Keywords:** dental implants, implantoplasty, tissue regeneration, citric acid, titanium

## Abstract

Peri-implantitis is an inflammatory disease caused by bacterial colonization that leads to progressive bone loss around dental implants. Implantoplasty is widely used for biofilm removal; however, it alters the titanium surface, generating particle release and impairing surface properties. This study evaluated whether a citric acid-based solution supplemented with collagen and magnesium cations could enhance hard and soft tissue regeneration following implantoplasty. Three surfaces were analyzed: physiological saline (Ctr), 25% citric acid (AC), and citric acid with collagen and magnesium nitrate hexahydrate (AC500/Mg). Surface roughness and wettability were assessed on titanium discs. Cytocompatibility, cell adhesion, and proliferation were evaluated using fibroblasts and osteoblasts up to 21 days, and mineralization was analyzed by alkaline phosphatase. In vivo studies were conducted in New Zealand rabbits with implants placed in the femur and muscle tissue. Surface roughness did not differ among treatments, while wettability significantly increased with citric acid-based solutions. All treatments showed good cytocompatibility. AC500/Mg significantly enhanced cell adhesion, proliferation, and osteoblast mineralization, showing threefold higher activity than controls at 21 days. In vivo, AC500/Mg exhibited greater bone contact (67%) and direct muscle integration, whereas AC and Ctr showed lower bone contact and fibrotic encapsulation. These results indicate that AC500/Mg improves soft and hard tissue responses without altering roughness, suggesting its potential as a regenerative strategy following implantoplasty.

## 1. Introduction

One of the major challenges currently associated with dental implants is their relatively high failure rate within the first 10 years of function, largely attributable to peri-implantitis. It is estimated that approximately 24% of placed dental implants must eventually be removed due to infection-induced bone loss [1,2,3]. When peri-implantitis is diagnosed, the standard clinical approach involves removal of the infected tissue, thorough decontamination of the affected area, placement of calcium phosphate-based biomaterials to promote bone regeneration, closure with soft tissue, and a healing period prior to the placement of a new implant [4,5,6].

This therapeutic sequence is highly time-consuming and represents a significant economic burden for the patient. Moreover, in certain cases, the regenerated space is insufficient for reimplantation, necessitating the sacrifice of an adjacent tooth. In response to these limitations, implantoplasty has emerged as an increasingly utilized technique for the removal of bacterial biofilms that develop on the surface of dental implants and abutments. Implantoplasty involves the mechanical modification of contaminated titanium surfaces using rotary instruments, typically diamond or tungsten carbide burs, to eliminate areas affected by bacterial colonization [7,8].

Despite its clinical utility, implantoplasty is associated with several drawbacks, including deterioration of the implant’s mechanical properties, reduced corrosion resistance, and loss of surface roughness, which is critical for bone tissue formation and subsequent mechanical and biological fixation [9,10,11]. However, the most significant concern is the release of titanium particles ranging in size from nanometers to several hundred micrometres. These particles have been shown to adversely affect the cytocompatibility of both fibroblasts and osteoblasts. Notably, nanometric particles often evade immune system detection, making their biological fate and long-term effects difficult to predict. In particular, Ti6Al4V particles have demonstrated pronounced cytotoxicity, underscoring the need for clinicians to meticulously aspirate or collect these particles from the implant site during implantoplasty procedures [12,13,14]. A systematic revision shows that the implantoplasty can be recommended as a budding treatment option for peri-implantitis, thus facilitating the reduction in the inflammatory reaction, followed by a high percentage success rate. Implantoplasty has been found to reduce the fracture resistance of standard diameter dental implants with external connection when there is a reduction in the body diameter of the implant [15].

Recent studies have shown that implantoplasty induces an inflammatory response in the surrounding tissues, leading to a localized decrease in oxygen concentration. This hypoxic environment selectively suppresses aerobic bacteria while allowing anaerobic bacteria to persist. Simultaneously, the increased metabolic demand of inflamed tissues promotes the reduction of titanium dioxide within the implant’s passive oxide layer, resulting in the exposure of metallic titanium at the implant surface, as oxygen is preferentially consumed by the tissues to prevent necrosis. As inflammation resolves, oxygen levels progressively recover, facilitating re-passivation of the titanium surface and the formation of new oxide layers. However, these newly formed layers are frequently non-stoichiometric mixed oxides, which have been reported to exhibit cytotoxic effects [15,16].

An additional concern is that implantoplasty significantly alters the implant surface topography, increasing surface energy and complicating the formation of new fibrotic or osseous tissue [17]. To mitigate this loss of biological activity, a mouthwash formulation based on citric acid, collagen, and divalent cations has been developed and shown to enhance the biological response of fibroblasts and osteoblasts. Citric acid further contributes to the formation of a stable passive layer on titanium, favouring the generation of titanium dioxide and preventing the formation of cytotoxic mixed oxides [18]. Additionally, the bactericidal properties of citric acid on titanium surfaces have been well documented [19,20,21,22].

In a previous study [23], different solutions capable of passivating titanium in the mouth were studied to prevent the toxicity of mixed titanium oxides produced by implantoplasty. This study also examined the biocompatibility of these solutions. The aim of this study is to examine in greater depth the biological behaviour in human fibroblasts and osteoblasts and their behaviour in vivo. Histologies surrounding the bone tissue, as well as around the animal’s muscle tissue, will confirm whether the treatment can regenerate bone and/or soft tissue to be applied to the titanium treated with implantoplasty and surrounding tissues. These in vitro findings have to be corroborated by in vivo experiments conducted in New Zealand rabbits. Dental implants were placed in the femur, and titanium cylinders were inserted into the muscle tissue. One group served as the control, while the experimental group received implants that had been immersed in the mouthwash solution prior to implantation.

The null hypothesis of the present study is that the mouthwash does not enhance the biological activity of fibroblastic or osteoblastic cells, nor does it promote bone or soft tissue regeneration around dental implants or abutments.

## 2. Materials and Methods

A total of 201 discs and 40 dental implants manufactured from commercially pure titanium (grade 3) were included in the present investigation. All specimens were provided by SOADCO S.L. (Klockner Medical Group, Escaldes Engordany, Andorra). Implantoplasty procedures were consistently performed by a single operator (JG) following a standardized drilling protocol. The process was carried out using a GENTLEsilence LUX 8000B dental turbine (KaVo Dental GmbH, Biberach an der Riß, Germany) with continuous water irrigation. Surface modification was conducted sequentially using fine-grit tungsten carbide burs (reference H379.314.014; KOMET GmbH and Co. KG, Lemgo, Germany).

Tungsten carbide burs were selected for the initial reshaping phase of the implant surfaces, with bur dimensions adapted according to the anatomical area treated. Larger-diameter burs were applied to vestibular and palatal regions, whereas burs with smaller diameters were reserved for areas with restricted access or interproximal zones. This instrumentation allowed for the effective removal of implant threads, resulting in a smoother surface topography. Final surface refinement was achieved through a polishing sequence employing silicon carbide polishers of decreasing abrasiveness. The polishing system consisted of a coarse-grit polisher (order no. 9608.314.030) followed by a fine-grit polisher (order no. 9618.314.030), both supplied by KOMET GmbH and Co. KG (Lemgo, Germany) [24].

Following surface treatment, all titanium discs were sterilized at 121 °C for 30 min. Subsequently, the specimens were immersed in three different chemical solutions, whose compositions are detailed in Table 1.

Citric acid treatments were selected based on the well-documented capacity of this compound to induce titanium surface passivation. The concentrations employed in this work were previously optimized and validated in earlier investigations [19,23,24]. The formulation containing collagen and magnesium nitrate hexahydrate was derived from a systematic optimization of different solution compositions and concentrations reported by Fernández Garrido et al. [23]. Aqueous solution with 25% by volume of citric acid and 10% by volume of Mg (NO_3_)_2_·6H_2_O. Add 0.5 g of collagen, which dissolves in the acidic medium of the citric acid. First, dissolve the collagen in the citric acid and add the magnesium salt to the aqueous medium. The final pH is 5.8.

### 2.1. Surface Roughness Characterization

Both smooth and micro-structured titanium surfaces were characterized using a white-light interferometric microscope (Wyko NT9300 Optical Profiler, Veeco Instruments, New York, NY, USA) operating in vertical scanning interferometry mode. For each experimental condition, 5 measurements were obtained from 3 independent discs for each treatment. Approximately 230 interferometric frames were captured per measurement, allowing high-resolution assessment of surface grooves. The analyzed surface areas comprised 127.7 × 95.8 µm for grooved regions and 63.1 × 47.3 µm for flat zones within the grooves. Post-processing and quantitative analysis were performed using Wyko Vision 4.10 software (Veeco Instruments, Plainview, NY, US), applying a Gaussian filter to correct for surface curvature and tilt. Surface roughness was expressed as the arithmetic mean roughness (Sa), defined as the mean of the absolute height deviations relative to the reference plane.

### 2.2. Wettability Assessment

Surface wettability was evaluated by determining the water contact angle (WCA) using the sessile drop technique. Three measurements were carried out on the 5 discs for each type of surface by depositing a 2 µL droplet of Milli-Q water and recording the contact angle with a goniometer (OCA 11, Dataphysics, Riverside, CA, USA). WCA values were obtained both before and after oxygen plasma treatment. For each surface, ten independent measurements were conducted under controlled conditions (37 °C and 100% relative humidity) to simulate physiological environments. Additionally, static contact angles of two reference liquids were measured following the same experimental protocol and environmental conditions used for water.

### 2.3. Fibroblast Culture and Viability Analysis

Primary human foreskin fibroblasts (HFFs; Millipore, Billerica, MA, USA) were maintained in phenol red-free Dulbecco’s Modified Eagle Medium (DMEM; Invitrogen, Carlsbad, CA, USA) supplemented with 10% fetal bovine serum (FBS), 2 mM L-glutamine, and penicillin/streptomycin (50 U/mL and 50 µg/mL, respectively). Cultures were incubated at 37 °C in a humidified atmosphere containing 5% CO_2_, with medium renewal every 48 h. Cells from passages 6 to 10 were used for all experiments.

For seeding, subconfluent cultures were enzymatically detached, centrifuged, and resuspended in serum-free, phenol red-free DMEM. Cells were seeded at a density of 6 × 10^3^ cells per disc onto micro-grooved titanium samples placed in 48-well plates coated with an agarose layer to prevent cell adhesion to the plastic substrate. Tissue culture polystyrene (TCPS) and polished commercially pure titanium discs served as reference materials. Cellular responses were evaluated at 4, 24, and 72 h post-seeding.

Cell adhesion and proliferation were quantified using the WST-1 colorimetric assay (Roche Applied Science, Penzberg, Germany), which measures mitochondrial metabolic activity via the conversion of tetrazolium salts into soluble formazan. At each time point, samples were incubated for 2 h with a 1:10 dilution of WST-1 reagent in serum-free DMEM. Absorbance was recorded at 440 nm using an ELx800 Universal Microplate Reader (Bio-Tek Instruments, Winooski, VT, USA). Measurements were conducted in triplicate for each surface across 7 independent discs. A calibration curve was generated using cell concentrations ranging from 3 × 10^3^ to 50 × 10^3^ cells.

Cytotoxicity was assessed by quantifying lactate dehydrogenase (LDH) release into the culture medium. Cell-free supernatants were collected, centrifuged at 250× *g* for 5 min, and analyzed using the Cytotoxicity Detection Kit (LDH, Roche Applied Science) following the manufacturer’s instructions. LDH activity was determined by measuring formazan formation at 490 nm. TCPS samples were used as the low cytotoxicity control, whereas lysed cells served as the high control. Analyses were performed on three samples per condition in two separate experiments. A total of 7 discs were used for this test for each surface studied.

### 2.4. Osteoblast Culture and Differentiation

Osteoblastic SaOS-2 cells, an epithelial-like human osteosarcoma-derived cell line, were employed for adhesion, proliferation, and differentiation assays. Cells were expanded for six to seven passages prior to experimentation, with routine monitoring of growth and viability. Cryopreserved cells were thawed in a 37 °C water bath, transferred to culture medium under sterile conditions, centrifuged, and resuspended before seeding into F175 culture flasks. Cultures were maintained at 37 °C in a 5% CO_2_ atmosphere, with medium replacement two to three times per week. A total of 7 discs were used for this test for each surface studied and for each test time.

The culture medium consisted of McCoy’s 5A Modified Medium supplemented with 1.5 mM L-glutamine, 2200 mg/L sodium bicarbonate, 15% FBS, 1% penicillin/streptomycin, and 2% sodium pyruvate. Cells were subcultured at approximately 90% confluence using 0.05% trypsin. Cell concentration and viability were determined using trypan blue exclusion and counting in a Neubauer chamber under phase-contrast microscopy.

Cell proliferation and metabolic activity were assessed using the resazurin-based Alamar Blue assay. A stock solution of resazurin (5 mg/mL) was prepared in PBS, diluted to a final concentration of 10 µg/mL in culture medium, and protected from light. After 3 days of culture, samples were washed with pre-warmed PBS and incubated with the resazurin solution for 3 h at 37 °C and 5% CO_2_. Aliquots were transferred to a 96-well plate, and absorbance was measured at 570 and 600 nm using an Infinite^®^ 200 PRO multimode microplate reader (TECAN).

Osteoblastic differentiation was evaluated by quantifying alkaline phosphatase (ALP) activity using a pNPP-based colorimetric assay (SensoLyte^®^, Anaspec, Fremont, CA, USA). Absorbance was recorded at 495 nm with an ELx800 microplate reader (Bio-Tek Instruments). A total of 7 discs were used for the ALP test at 21 days for each treatment.

### 2.5. In Vivo Evaluation

In vivo experiments were conducted using 10 healthy adult New Zealand white rabbits (6–7 months old; mean body weight ~5 kg) obtained from the Universitat Autònoma de Barcelona (Cerdanyola, Spain). All procedures were approved by the institutional ethics committee (approval code CEEH2013-05) and performed at the Animal Experimentation Facility of the Universitat Autònoma de Barcelona in accordance with Spanish and European regulations for animal care and use. Animals were housed in enriched cages permitting normal activity and were monitored daily by trained personnel. Food and water were provided ad libitum. Detailed ethical approvals and institutional authorizations are included in the Supplementary Materials.

#### 2.5.1. Implants

The implants were placed in the rabbits’ femurs in a double-blind procedure. Two implants were placed in each femur, making a total of 40 implants. Each animal received two implants in the right and left femur, and 40 implants in 10 animals. The experimental site was located on both distal lateral condyles of the femur. A total of 13 control implants, 13 implants treated with citric acid, and 14 implants treated with citric acid, collagen and dissolved magnesium salt were implanted. They were placed blindly, and the histology studies were performed in the same manner.

#### 2.5.2. Surgical Procedures

All surgical procedures were performed in an operating room under sterile conditions, and under general anesthesia induced and maintained on a concentration of 2.5–4% of isoflurane (Isoba-vet, Schering-Plough, Madrid, Spain). The animals were first sedated with a combination of medetomidine (50 mg/kg/i.m., Domtor, Esteve, Barcelona, Spain) and ketamine (25 mg/kg/i.m., Imalgène 1000, Merial, Toulouse, France). During anesthesia, the animals were continuously monitored by a veterinarian, category B or C.

The animal received antibiotic prophylaxis during one week with enrofloxacin (15 mg/Kg/s.c. once a day, Ganadexil 5%, Invesa, Barcelona, Spain) and pain was controlled with meloxicam (0.2 mg/Kg/s.c., Metacam, Boehringer Ingelheim, Barcelona, Spain) during three days.

**Figure 1:**
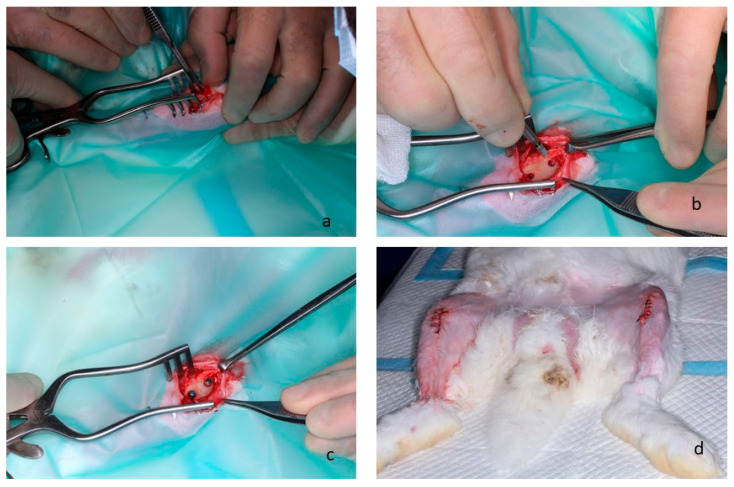
(**a**) Cut with a scalpel to observe the bone where the implants will be placed, and use the drill to make the two holes for implanting the implants. (**b**) Two holes were made for placing the implants. (**c**) Implants were placed in the distal femurs. (**d**) Sutures on the rabbits’ legs and disinfection.

In Figure 1, the in vivo surgery details can be observed. After three weeks of quarantine, the animals were induced into general anesthesia. After shaving and disinfecting, the femoral condyles were exposed by a lateral longitudinal incision. Implant bed preparations were carried out according to the recommendations of the manufacturers. Finally, the muscle, the subcutaneous tissue and the skin were sutured in layers with reabsorbable sutures (Vicryl 4-0, Ethicon, Raritan, NJ, USA).

A total of 21 days later, rabbits were painlessly sacrificed by a sodium pentobarbital overdose (100 mg/Kg/i.v., Dolethal, Vétoquinol, Madrid, Spain) after sedation with ketamine and medetomidine.

### 2.6. Histological and Histomorphometric Evaluation

Bone segments containing the implant, together with the surrounding hard and soft peri-implant tissues from the distal distracted femur, were harvested using an oscillating saw. The retrieved specimens were fixed, labelled, and subsequently dehydrated through a graded ethanol series ranging from 50% to 100%. Dehydrated samples were then infiltrated with progressively increasing concentrations of glycometacrylate resin (Technovit 7200 VLC, Heraus Kulzer, Werheim, Germany) diluted in ethanol, following established protocols described previously [25]. Polymerization was carried out at 37 °C for 24 h to ensure complete resin curing.

Central longitudinal sections of the implants were obtained using a precision band saw. The resulting sections, initially approximately 200 µm thick, were mechanically ground and polished (Exakt Apparatebau, Norderstedt, Germany) using silicon carbide abrasive papers with grit sizes of 1200 and 4000 (Struers, Copenhagen, Denmark) until a final thickness of about 40 µm was achieved. Histological staining was performed according to the Levai–Laczkó technique [26], allowing both qualitative histological observation and quantitative histomorphometric measurements.

Histological and histomorphometric analyses were conducted blindly using a motorized light microscope equipped with a digital imaging system (BX51 microscope coupled with DP71 camera; Olympus Corporation, Tokyo, Japan). The peri-implant region was defined as the area of interest for all evaluations. Image processing and tissue discrimination were based on differences in colour and morphology, enabling identification of newly formed bone, lamellar bone, connective tissue, and vascular structures (Adobe Photoshop, San Jose, CA, USA). All measurements were performed by a blinded examiner using dedicated image analysis software vesrion 4.0 (cellSens 1.5, Olympus Corporation, Tokyo, Japan). Bone-to-implant contact (BIC) was calculated as the percentage of the implant surface directly apposed to mineralized bone.

The implants placed in the rabbit’s muscle area were examined to determine the formation of new tissue around the implant and the possible presence of a fibrotic capsule around the implant, or whether there was direct muscle tissue growth on the titanium cylinder. The presence of granulomatous tissue and inflammation in the tissues around the implant was also studied.

### 2.7. Number of Samples

Roughness: 3 discs × 3 treatments = 6 discs

Wettability: 3 discs × 3 treatments = 6 discs

Fibroblasts: 7 discs × 3 treatments × 3 times = 63 discs

Citocompatibility: 7 discs × 3 treatments = 21 discs

Osteoblasts: 7 discs × 3 treatments × 4 times = 84 discs

ALP activity: 7 discs × 3 treatments × 1 times = 21 discs

In vivo: 10 rabbits × 2 femurs/rabbit × 2 implants/femur = 40 implants (13 control, 13 CA and 14 AC500/Mg.

TOTAL: 201 discs and 40 implants

### 2.8. Statistical Analysis

Statistical analyses were performed using Student’s *t*-test, one-way analysis of variance (ANOVA), and Tukey’s post hoc multiple comparison test to identify significant differences among experimental groups. Statistical significance was set at *p* < 0.05.

## 3. Results

**Figure 2:**
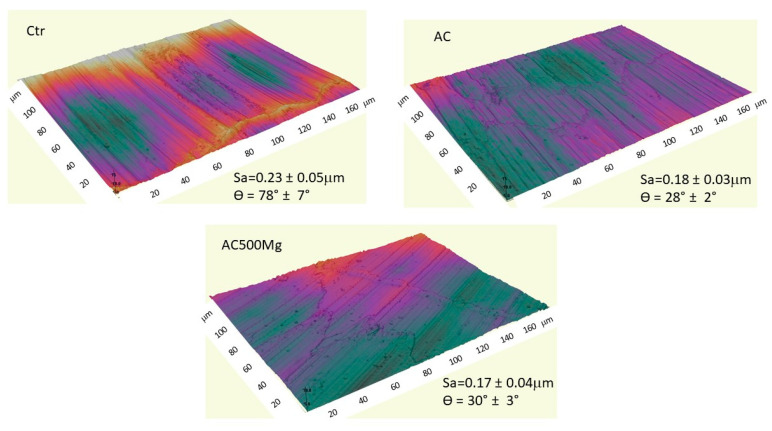
Confocal microscopy images of the different surfaces studied for the three types of surfaces. For each of them, the roughness is described in terms of Sa values and the contact angles obtained.

The results of roughness determined by confocal microscopy can be seen in Figure 2, where the Sa parameter is shown. It can be seen that the control samples have a higher roughness than those treated with acid solutions, as the acid rounds off the peaks and valleys, slightly reducing roughness. The same figure describes the contact angles, showing that the control sample is the most hydrophobic at 78°, as well as the increase in wettability for the samples treated with both citric acid-based solutions.

**Figure 3:**
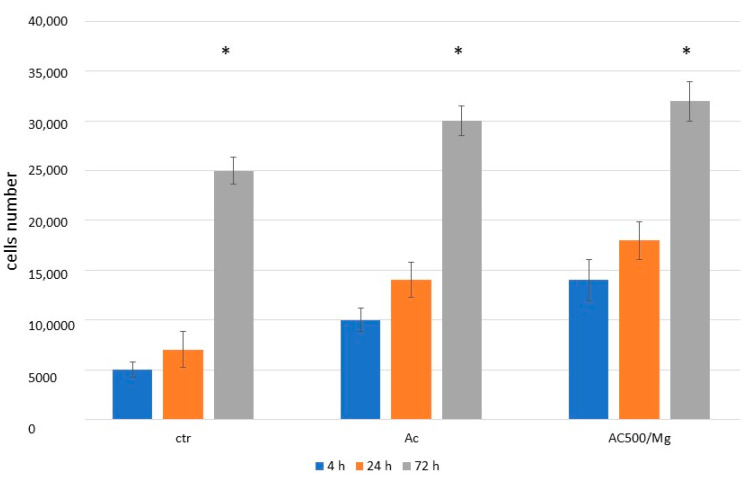
Number of fibroblasts at different seeding times for the three surfaces studied. The asterisks show statistically significant differences between the different times for each type of treatment.

The study of fibroblasts is shown in Figure 3, showing the adhesion of these cells to the different surfaces studied. It can be observed that the surfaces treated with citric acid show an increase in the number of fibroblasts compared to the control. However, the surface containing collagen and divalent magnesium cations shows statistically significant differences with a *p* < 0.05.

**Figure 4:**
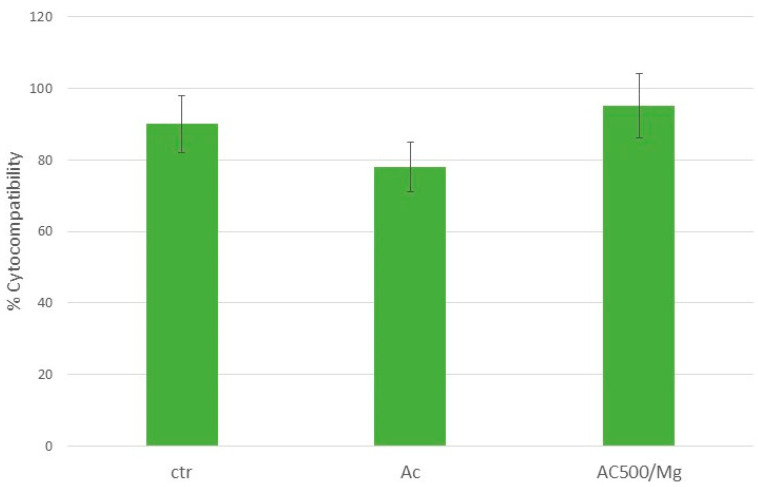
Percentage of cytocompatibility of the three surfaces studied.

These results show the excellent adhesion of the surface, especially the citric acid solution with collagen and divalent cations, which may indicate good performance in the regeneration of soft tissue around the titanium surface. Likewise, the degree of cytocompatibility of the solutions has been determined with respect to the control surface that has not been treated with citric acid or with the citric acid and collagen solution with divalent magnesium ions. Figure 4 shows that the three surfaces have cell survival values above 70%, which is the limit of cytotoxicity.

**Figure 5:**
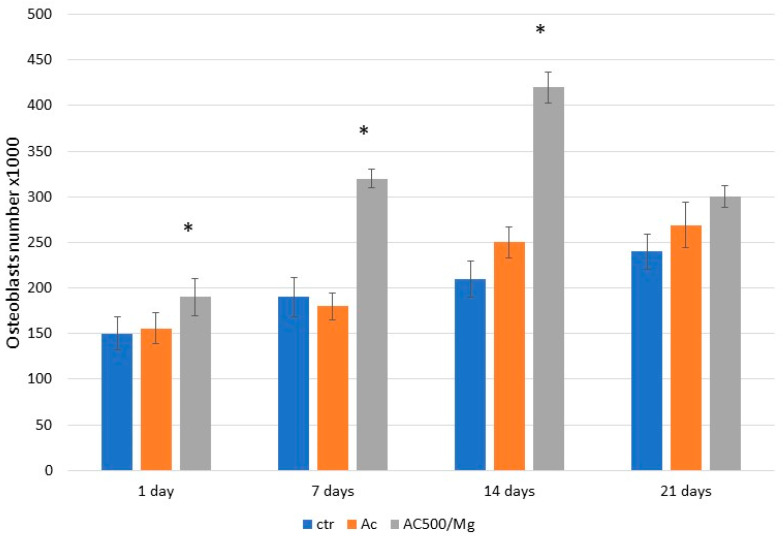
Number of osteoblast cells on the surfaces studied after different periods of time. The asterisks show statistically significant differences between each type of surface with *p* < 0.05.

**Figure 6:**
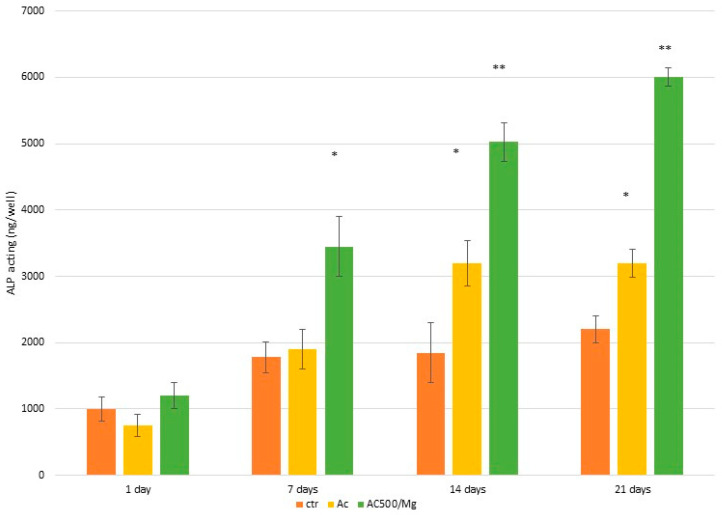
Alkaline phosphatase levels (cell mineralization) for the three surfaces studied. The asterisks show statistically significant differences between each type of surface. The double asterisk means that there are statistically significant differences between the columns without and with a single asterisk.

Figure 5 shows the adhesion of osteoblasts on the three surfaces at different times. The best performance can be seen with the citric acid solution with collagen and divalent magnesium cations, where statistically significant differences are shown with *p* < 0.005 for the times of 1, 7, and 14 days. However, at 21 days, adhesion decreases significantly compared to 14 days, due to the fact that the cells have already adhered completely and are mineralizing. This can be seen by observing the alkaline phosphatase values in Figure 6, where the alkaline phosphatase values are much higher in the AC500/Mg solution.

**Figure 7:**
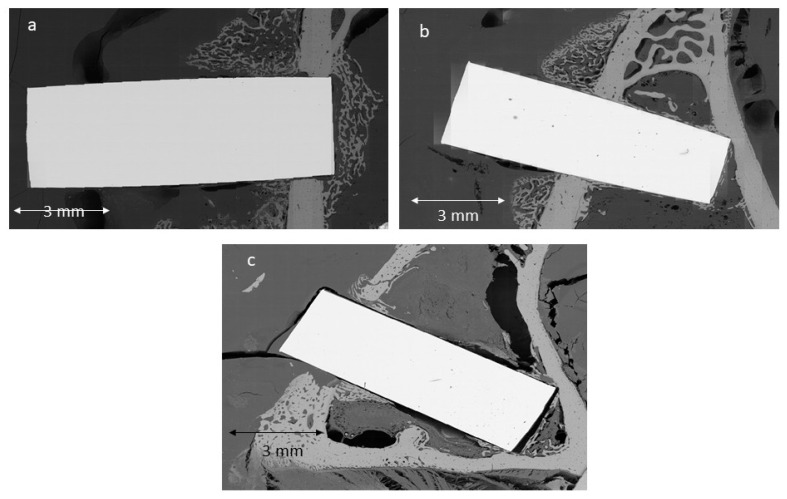
Histologies observed using scanning electron microscopy (**a**) control cylinder, (**b**) cylinder treated with AC, and (**c**) cylinder treated with AC500/Mg.

**Figure 8:**
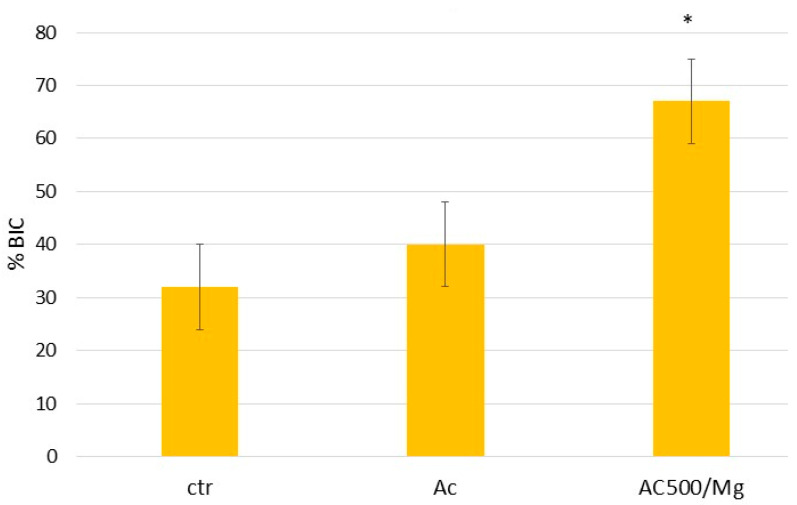
Bone implant contact after 21 days of implantation. The asterisk shows statistically significant differences between each type of surface with *p* < 0.05.

Figure 7 shows images obtained by tilting in scanning electron microscopy of cylinders with the three surfaces studied. Greater bone-implant contact (BIC) can be seen on the AC500 Mg surface. The BIC results can be seen in Figure 8.

**Figure 9:**
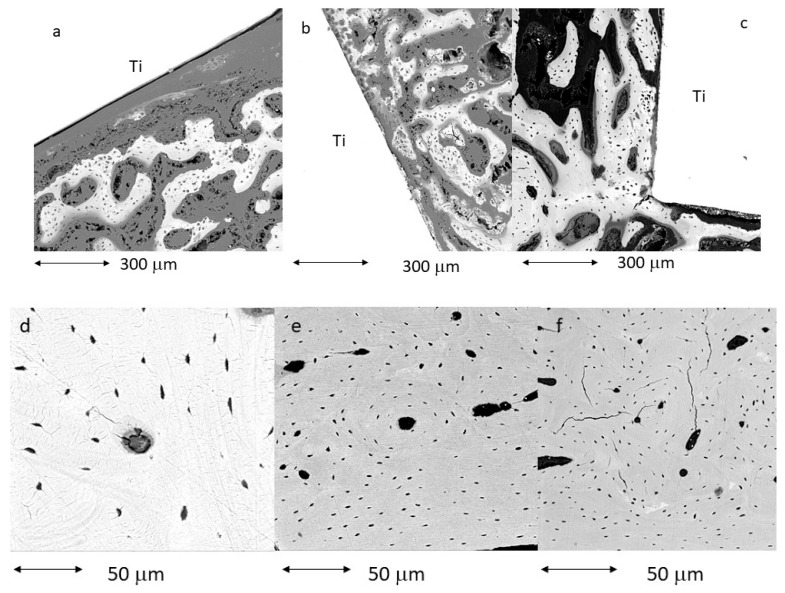
Histologies of cortical bone formed around the implant. (**a**) control. (**b**) ac. (**c**) AC500/Mg. (**d**) at higher magnification for control. (**e**) at higher magnification for Ac. (**f**) at higher magnification for AC500/Mg.

In Figure 9, the bone tissue formed around the implant can be observed at higher magnification. In all cases, new cortical bone has grown on the titanium surface without soft tissue in any case.

**Figure 10:**
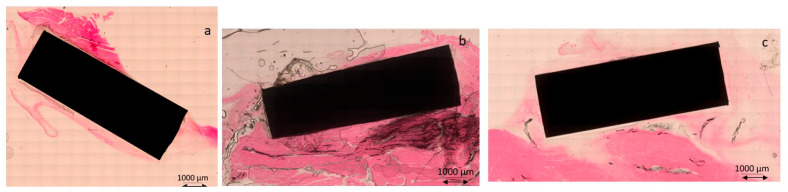
Histologies for the cylinders implanted in muscular tissue. (**a**) control. (**b**) ac. (**c**) AC500/Mg.

Figure 10 shows the histologies in the muscle tissue area, where the control and AC samples show the formation of a fibrotic capsule on the surface of the titanium, but in the cylinders treated with AC500/Mg, there is no formation of this capsule.

**Figure 11:**
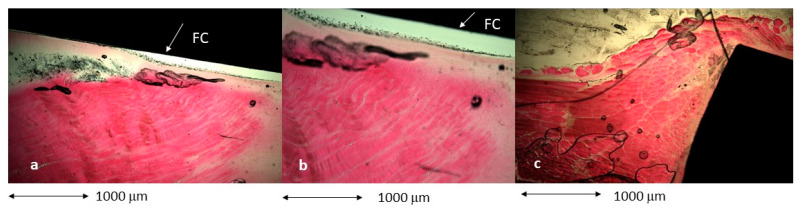
Histologies for the cylinders implanted in muscular tissue. (**a**) control. (**b**) ac. (**c**) AC500/Mg at higher magnification.

At higher magnification, the histologies from Figure 11 are shown, where the appearance of a fibrotic capsule around the titanium can be observed in Ctr and AC treatments. The sample treated with AC500/Mg does not show the fibrotic capsule; the contact of titanium with the muscular tissue is direct (Figure 11).

**Figure 12:**
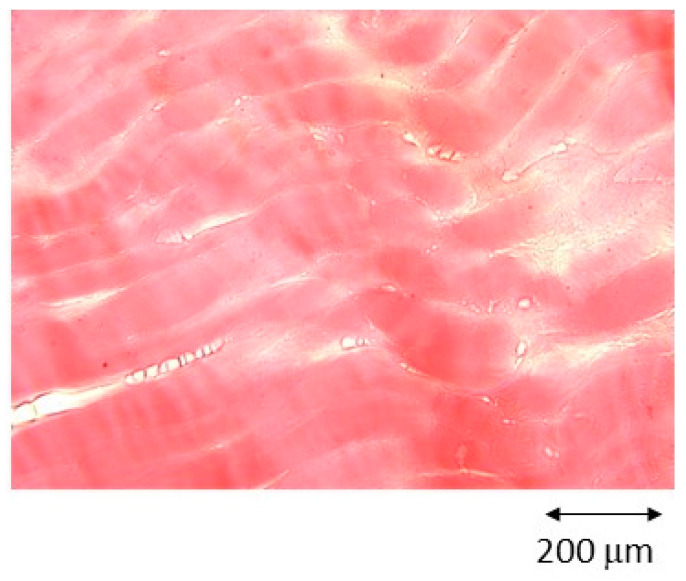
Histology of muscle tissue formed around the treated AC500/Mg cylinder, where the striations characteristic of muscle tissue can be observed. No irregular tissue or tumours produced by giant cells are visible.

No abnormal inflammatory responses or discomfort were observed in the animals at the three implant sites, and the rabbits’ mobility was adequate. Histology revealed growth of striated muscle tissue next to the implant, indicating good integration into the soft tissue (Figure 12).

## 4. Discussion

Citric acid was used in the solution for this dental mouthwash because numerous authors have demonstrated that citric acid is biocompatible as a mild acid and allows titanium oxide passivation layers approximately 6 nanometers thick to be obtained with a homogeneous and compact morphology, without porosity, which prevents the release of metal ions into the physiological environment and maintains good corrosion resistance results. In addition to these physicochemical properties, several authors have demonstrated the bactericidal capacity of citric acid against Gram + and Gram – bacterial strains [18,19,20]. Therefore, the titanium surface that has undergone implantoplasty and has been able to generate mixed oxides with this treatment will undergo repassivation, and a totally biocompatible titanium dioxide layer will be obtained on the surface.

It is important that the base of this mouthwash is acidic, as this allows us to dissolve the collagen in the solution and thus incorporate it into the physiological medium. We have also observed that treatment with citric acid-based solutions increases wettability, as the control surface has a contact angle of 78°, and after treatment with the citric acid solution, the values are reduced to between 28 and 30°. This increase in wettability is a very positive factor for biological activity, as better wettability means that there is more contact between the blood and the titanium surface and, therefore, a greater number of proteins will be adsorbed by the surface, leading to greater cell migration [27,28,29]. This can be seen in the increase in fibroblast and osteoblast cells adhering to the surface and how they proliferate over time. We can observe that after 21 days, there is no further growth in the number of cells, and this is because at that time the cells are already differentiating into soft or bone tissue [30,31,32].

The high degree of compatibility shown according to the ISO standard [33] for the citrus solutions studied is noteworthy, which provides assurance of the biocompatibility of the solutions studied. It is important to note that high levels of alkaline phosphatase are observed in osteoblast cells, reflecting the good health of osteoblast cells in the differentiation process [34,35]. This increase in phosphatase is statistically significant in the solution containing divalent magnesium cations. The beneficial action of divalent cations such as Ca and Mg is well known in bone regeneration, which is why there are numerous bone cements based on calcium phosphates and other regeneration materials containing these cations, which act as true catalysts for regeneration [34,35,36,37,38,39,40]. In terms of re-osseointegration

In terms of osseointegration, the following should be taken into account: various implant surface decontamination techniques, chemical and/or mechanical, have been used either alone or simultaneously with/without guided bone regeneration (GBR). Despite the access-flap surgery, it was observed that application of a single decontamination measure, either chemical or mechanical, was not adequate to provide a better treatment outcome. Furthermore, promising results were observed in the studies that used a combination of bone graft or bone substitutes together with GBR for the regenerative therapy. In terms of implant surfaces, better re-osseointegration was observed with rough implant surfaces when combined with GBR. The treatment of periimplantitis is not predictable; however, surgical treatment could be considered the most anticipated treatment option when combined with chemical and mechanical means for implant surface decontamination. Laser therapy is a recent modality that needs more exploration to identify the best beneficial laser parameters that can be used [41]. In addition, new surfaces with structures that promote osteointegration are being studied to accelerate tissue regeneration [42,43,44].

This good cellular behaviour is reflected in good in vivo behaviour when titanium cylinders treated with citric acid, collagen, and magnesium salts achieve more than 60% bone tissue when implanted in rabbit femurs. It can be observed that citric acid improves the bone index contact by almost 10%, probably due to the greater wettability provided by the citric acid treatment, but the results do not show statistically significant differences. Histology shows that, in all three cases, the bone grows in close contact with the titanium without the appearance of fibrotic capsules between the newly formed bone tissue and the titanium implant.

However, in the titanium cylinders implanted in rabbit muscle tissue, it can be seen that the control cylinders and those treated with citric acid show a fibrotic capsule between the muscle tissue and the titanium cylinder, demonstrating that in these cases, there has been an immune response to the implant [42,43]. However, in the cylinders treated with citric solution, collagen, and divalent magnesium cations, no fibrotic capsule is observed, and histology shows the growth of orderly and well-differentiated muscle tissue from the titanium. It has also been observed that there has been no inflammatory reaction, as there is no evidence of giant cells or alterations in tissue morphology [44,45].

Currently, the methods used by clinicians for bone regeneration in implantoplasty procedures involve placing calcium phosphates, generally in the form of granules [46,47,48,49]. This methodology has its problems, as the granules sometimes move around the cavity and do not remain in the regeneration area. For this reason, membrane systems are used to retain the calcium phosphates. Different types of phosphates are used to accelerate bone formation. In principle, if they are small in size, formation is faster than when the granules are larger. Regeneration processes can range from 7 to 15 months, and in some cases, bone formation on the surface of the granule prevents blood from reaching the central areas of the particles, resulting in incomplete regeneration of the biomaterial [50,51,52]. To reduce osseointegration times, collagen has been added to the phosphates, creating a composite material that promotes re-osseointegration. In the case of soft tissues, there are not as many products available, as clinicians focus primarily on bone regeneration because it is the bone that mechanically fixes the implant and thus gives the dental implant its functionality. There are some products based on hyaluronic acid and chitosan that show promising results [52,53]. Our contribution is that AC500/Mg mouthwash not only promotes the regeneration of the tissues surrounding the implant but also allows the passivation of the titanium affected by implantoplasty to prevent possible cytotoxicity. The acidic citric environment dissolves collagen, and the divalent cations activate the formation of hard tissue.

A biomimetic surface has been achieved in titanium that manages to passivate it and inhibit the immune response and bone and muscle tissue growth around the treated implant, without inflammatory or immune reactions. Therefore, this solution offers hope for dental implant treatments, as it aids tissue formation in addition to its known bactericidal properties. Its application after implantoplasty treatments following peri-implantitis may be particularly interesting, not only for tissue regeneration, but also for the re-passivation of titanium, which may have been altered by the mechanization of titanium and the inflammation caused in the tissues.

This research has some limitations that must be considered, such as the animal model and the implantation site; the conditions of the rabbit femur are not the same as those of the oral cavity, nor do they have the same mechanical loads. It is also well known that rabbit bone has cortical bone but very little cancellous bone, and therefore, the bone index contact values are lower than those that would be seen with cortical and cancellous bone. In any case, it is also known that bone formation metabolism in rabbits is faster than in humans [41,54,55,56,57,58,59]. The work should be completed with microbiological studies using the new mouthwash and biofilm formation to determine the bactericidal capacity of citric acid treatment with this new solution. With the experimental results obtained both in vitro and in vivo, we have been able to verify the falsity of the proposed null hypothesis.

## 5. Conclusions

The citric acid solution with collagen and divalent magnesium cations makes the titanium surface more hydrophilic than the control, improving the adhesion, proliferation, and differentiation values of human fibroblasts and osteoblasts at the different times studied. A higher level of alkaline phosphatase was observed after 21 days of SaOS-2 osteoblast culture, indicating a significant improvement in hard tissue mineralization. In vivo studies show bone index contact values close to 70%, much higher than the other treatments, which reach values from 33 to 40%. It has been demonstrated that treatment of titanium with AC500/Mg in muscle tissue does not form a fibrotic capsule between the newly formed tissue and the titanium, unlike the other treatments studied. These results provide a solution that allows for the regeneration of both muscle and bone tissue and offers hope for clinical improvement in patients who have undergone implantoplasty.

## Figures and Tables

**Table 1:** Chemical compositions of the solutions studied.

Samples	Chemical Composition
Control	Saline solution (0.9% NaCl in volume)
25% Citric acid (AC)	25% volume aqueous solution of citric acid.
25% Citric acid + Collagen 500 + 10% Mg (AC 500/Mg)	Aqueous solution containing 25% of citric acid and 10% of Mg (NO_3_)_2_·6H_2_O. The percentages are in volume. Add 0.5 g of collagen, which dissolves in the acidic medium of the citric acid.

## Data Availability

The original contributions presented in the study are included in the article, further inquiries can be directed to the corresponding author.

## Supplementary Materials

The following supporting information can be downloaded at: https://www.mdpi.com/article/10.3390/biomimetics11020116/s1.
