# Supplementary material for: Tissue Regeneration on Implantoplasty-Treated Implants Using a Citric Acid–Collagen–Magnesium-Based Solution: An In Vitro and In Vivo Study
Source: Biomimetics (Basel). 2026 Feb 4;11(2):116. doi: 10.3390/biomimetics11020116 (PMC12938264; doi:10.3390/biomimetics11020116)
Supplement: Supplementary file 1 [file biomimetics-11-00116-s001.zip › biomimetics-4103899-supplementary.pdf]

Vicerectorat de Recerca i de Transferència

Universitat Autònoma de Barcelona

Plaça Acadèmica
Edifici A – Campus de la UAB
08193 Bellaterra (Cerdanyola del Vallès)
Barcelona – Spain
Tel. +34 93 581 35 78
ceeah@uab.cat
http://www.uab.cat/etica-recerca/

ETHICS COMMITTEE ON ANIMAL AND HUMAN EXPERIMENTATION (CEEAH) OF THE UNIVERSITAT AUTÒNOMA DE BARCELONA

Pursuant to the ethical and legal requirements (5/1995/*Generalitat de Cataluña*, 214/1997/*Generalitat de Cataluña*, Spanish Royal Decree 53/2013, 86/609/EEC, 91/628/EEC, 92/65/EEC, Directive 2010/63/EU) regarding the use of animals in research, the Ethics Committee on Animal and Human Experimentation (CEEAH) of the Universitat Autònoma de Barcelona reports that the methodologies involving the use of experimental animals at the UAB, in the project **Aplicació quirúrgica de nous implants quirúrgico per avaluar-ne la seva osteointegració i resposta tisular** are described in the procedure with reference number:

- **CEEAH 2016: Aplicació quirúrgica de nous implants quirúrgico per avaluar-ne la seva osteointegració i resposta tisular** approved in the minutes number 2013-05 of 04-26-2013

which, on submission to this Committee, has been issued with a favourable report.

Pursuant to Sections a), b), c) and d) of Article 28 of Decree 214/1997, of 30 July, on the use of animals in experiments and for other scientific purposes, (DOGC: Catalan Official Government Bulletin no. 2450 of 7 August 1997), the CEEAH has evaluated the aforementioned protocol on the following criteria.

- Suitability of the procedure with regard to the objectives of the study, possibility of reaching valid conclusions with the lowest possible number of animals, consideration given to alternative methods not requiring the use of animals and suitability of the species chosen.

- Measures in place to avoid unnecessary suffering for the animals and, where necessary, provide analgesics, anaesthetics or other methods of eliminating pain, suffering and distress as much as possible.

- Use of humane euthanasia methods.

- Level of training of personnel carrying out tasks within the procedure.

JOSE LUIS MOLINA GONZALEZ - DNI 36561625C

Firmado digitalmente por JOSE LUIS MOLINA GONZALEZ - DNI 36561625C
Fecha: 2022.02.15 09:24:07 +01'00'

José Luis Molina
President of the Ethics Committee (CEEAH)

Bellaterra (Cerdanyola del Vallès), February 15th 2022
